# Lansoprazole for secondary prevention of gastric or duodenal ulcers associated with long-term non-steroidal anti-inflammatory drug (NSAID) therapy: results of a prospective, multicenter, double-blind, randomized, double-dummy, active-controlled trial

**DOI:** 10.1007/s00535-012-0541-z

**Published:** 2012-03-03

**Authors:** Kentaro Sugano, Teiji Kontani, Shinichi Katsuo, Yoshinori Takei, Nobuhiro Sakaki, Kiyoshi Ashida, Yuji Mizokami, Masahiro Asaka, Shigeyuki Matsui, Tatsuya Kanto, Satoshi Soen, Tsutomu Takeuchi, Hideyuki Hiraishi, Naoki Hiramatsu

**Affiliations:** 1 Division of Gastroenterology, Department of Medicine, Jichi Medical University, 3311-1 Yakushiji, Shimotsuke, Tochigi 329-0498 Japan; 2 Komatsu Municipal Hospital, Ho 60, Mukaimoto-Orimachi, Komatsu, Ishikawa 923-0961 Japan; 3 Fukui General Hospital, 1-42-1 Nittazuka, Fukui, Fukui 910-0067 Japan; 4 Izumino Hospital, 2-10-53 Azouno-kitamachi, Kochi, Kochi 781-0011 Japan; 5 Foundation for Detection of Early Gastric Cancer, 2-6-12 Nihonbashi-Kayabacho, Chuoku, Tokyo, 103-8790 Japan; 6 Department of Gastroenterology, Osaka Saiseikai Nakatsu Hospital, 2-10-39 Shibata, Kita-ku, Osaka, 530-0012 Japan; 7 Department of Gastroenterology, Tsukuba University Hospital, 2-1-1 Amakubo, Tsukuba, Ibaraki 305-8567 Japan; 8 Department of Cancer Preventive Medicine, Graduate School of Medicine, Hokkaido University, Kita 2, Nishi 7, Kita-ku, Sapporo, 060-0812 Japan; 9 Department of Data Science, The Institute of Statistical Mathematics, 10-3 Midorimachi, Tachikawa, Tokyo 190-8562 Japan; 10 Department of Gastroenterology and Hepatology, Osaka University Graduate School of Medicine, Yamadaoka, Suita, Osaka 565-0871 Japan; 11 Nara Hospital Kinki University Faculty of Medicine, 1248-1 Otodacho, Ikoma, Nara 630-0227 Japan; 12 Division of Rheumatology, Department of Internal Medicine, Keio University School of Medicine, 35 Shinanomachi, Shinjuku, Tokyo, 160-8582 Japan; 13 Department of Gastroenterology, Dokkyo Medical University, 880 Kita-Kobayashi, Mibu-machi, Shimotsuga-gun, Tochigi, 321-0207 Japan

**Keywords:** Non-steroidal anti-inflammatory drugs, Rheumatoid arthritis, Osteoarthritis, Gastric or duodenal ulcers, Prevention, Lansoprazole, Active-controlled trial

## Abstract

**Background:**

Low-dose lansoprazole has not been intensively evaluated for its efficacy in the prevention of recurrent gastric or duodenal ulcers in patients receiving long-term non-steroidal anti-inflammatory drug (NSAID) therapy for pain relief in such diseases as rheumatoid arthritis, osteoarthritis, and low back pain.

**Methods:**

This multi-center, prospective, double-blind, randomized, active-controlled study involving 99 sites in Japan was designed to compare the efficacy of lansoprazole (15 mg daily) with gefarnate (50 mg twice daily). Patients with a history of gastric or duodenal ulcers who required long-term NSAID therapy were randomized to receive lansoprazole 15 mg daily (*n* = 185) or gefarnate 50 mg twice daily (*n* = 181) and followed up for 12 months or longer prospectively.

**Results:**

The cumulative incidence of gastric or duodenal ulcer at days 91, 181, and 361 from the start of the study was calculated by the Kaplan–Meier method as 3.3, 5.9, and 12.7%, respectively, in the lansoprazole group versus 18.7, 28.5, and 36.9%, respectively, in the gefarnate group. The risk for ulcer development was significantly (log-rank test, *P* < 0.0001) lower in the lansoprazole group than in the gefarnate group, with the hazard ratio being 0.2510 (95% CI 0.1400–0.4499). A long-term follow-up study showed an acceptable safety profile for low-dose lansoprazole therapy, with diarrhea as the most frequent adverse event.

**Conclusion:**

Lansoprazole was superior to gefarnate in reducing the risk of gastric or duodenal ulcer recurrence in patients with a definite history of gastric or duodenal ulcers who required long-term NSAID therapy.

**Electronic supplementary material:**

The online version of this article (doi:10.1007/s00535-012-0541-z) contains supplementary material, which is available to authorized users.

## Introduction

Non-steroidal anti-inflammatory drugs (NSAIDs) continue to be in widespread use due to an increase in the prevalence of diseases in the aging population that respond to NSAIDs and due to their crucial role as effective antipyretic analgesics in a wide spectrum of conditions and diseases ranging from a common cold to rheumatoid arthritis (RA). However, they are known to disrupt the mucosal resistance to gastric acid through mechanisms including suppression of prostaglandin production in the gastric mucosa, and are thus associated with adverse events such as gastric or duodenal ulcers.

In a meta-analysis published in 1991 [1], the overall odds ratio (OR) of the risk for adverse gastrointestinal (GI) events in NSAID users was shown to be 2.74 compared to non-NSAID users based on data from 16 studies, with this relative risk markedly increased among high-risk patients, i.e., those with additional risk factors such as an age greater than 60 years, a previous history of GI events, and concomitant corticosteroid use [1–3].

Various guidelines recommend discontinuation of NSAID therapy at the onset of GI events, such as GI bleeding [4]. However, NSAID users include patients who require long-term NSAID therapy, such as those with RA, and discontinuation of NSAID therapy in these patients is associated with decreased quality of life due to the return of pain and inflammation. Thus, it is vitally important to ensure continued prophylaxis of GI adverse effects associated with NSAID use in patients requiring long-term NSAID therapy.

In this context, a number of controlled studies have reported on the prevention of gastric or duodenal ulcers in patients during NSAID therapy with misoprostol, proton pump inhibitors (PPIs), and histamine H_2_ receptor antagonists (H_2_RAs) [5–11]. Based on the evidence obtained to date, a clinical expert consensus statement [4] recommends PPIs as preferred agents for the prophylaxis of NSAID-associated GI injury. However, to date, low-dose lansoprazole has not been evaluated in a clinical trial for its prophylactic efficacy in patients with definitive evidence of previous ulcer development with/without *Helicobacter pylori* (*H. pylori*) infection, although low-dose or regular-dose lansoprazole was shown to be effective in preventing NSAID-induced ulcers in patients without *H. pylori* infection [7, 9].

This study aimed to examine the preventive effect of low-dose lansoprazole (15 mg daily) against the recurrence of gastric or duodenal ulcers associated with long-term NSAID therapy excluding low-dose aspirin (LDA) in patients with definitive evidence of previous ulcer development, which is counted among distinct risk factors for GI bleeding. Ulcer recurrence was defined as endoscopically confirmed ulcers based on the predefined criteria and reconfirmed by an independent panel of endoscopists. The occurrences of gastric or duodenal bleeding requiring or not requiring hospitalization were also evaluated.

## Methods

### Design overview

The study protocol was approved by the ethics committee of each participating institution, and all patients gave written informed consent to participate in the study.

The Independent Data Monitoring Committee planned an interim analysis in advance to investigate whether or not to continue the study in light of interim efficacy and safety findings, based on the predefined criteria. However, the Independent Data Monitoring Committee recommended discontinuing this trial based on the final results of a companion trial of lansoprazole for prevention of gastric or duodenal ulcers associated with LDA therapy, which showed strong efficacy of low-dose lansoprazole [12]. After the Committee made the decision to discontinue the double-blind trial, the patients in the 47 healthcare institutions were invited to move on to the follow-up study with open-label lansoprazole treatment lasting up to 6 months. This trial was registered with ClinicalTrials.gov (number NCT00787254).

### Setting and participants

Patients were enrolled in the study if they met the following criteria: those who were taking an NSAID when they gave informed consent, and who required long-term NSAID therapy (LDA excluded) after the start of the study (day 1) with the investigational drug and those in whom a history of gastric and/or duodenal ulcer was confirmed by endoscopy, i.e., those who were confirmed to have an ulcer scar either on day 1 or through an endoscopic examination (e.g., photographs, films) performed prior to day 1.

Patients were excluded if they had an open gastric or duodenal ulcer or an active upper GI hemorrhage confirmed by endoscopy on day 1, aspirin-induced asthma or hypersensitivity to NSAIDs including aspirin or a history of hypersensitivity, a history of surgery or a planned operation that could affect gastric secretion (e.g., upper GI tract resection, vagotomy), clinically significant liver or kidney disorders [including liver tests demonstrating AST (GOT)/ALT (GPT) values 2.5 times or higher than the upper limit of normal or creatinine levels 2.0 times or higher than the upper limit of normal], severe cardiac dysfunction, hypertension, or hematological diseases, and active cancers.

All patients confirmed to be eligible at each trial site were reassessed for their eligibility, based on endoscopic images either on films or data submitted after randomization, by an independent panel of expert endoscopists.

### Randomization and intervention

Patients who met the inclusion criteria were randomly assigned to either of the following two treatment groups: a group receiving the investigational drug (lansoprazole 15 mg orally given once daily) and cytoprotective anti-ulcer agent gefarnate placebos (twice daily) or a group receiving gefarnate (50 mg orally given twice daily) and the lansoprazole placebo (once daily), in combination with an NSAID at the doses indicated in their package inserts for a duration of 6 months or longer (up to 24 months). Acetaminophen and celecoxib, which are reported to be less associated with GI injury, were excluded, along with LDA, which was studied in a separate trial [12]. Lansoprazole and gefarnate placebos were used to ensure that all patients followed the same regimen and that blinding was maintained. Treatment groups were assigned by using computer-generated random sequence numbers. Patients were randomly assigned by investigators to receive lansoprazole or gefarnate in a 1:1 ratio according to the unique sequential numbers for the study drugs, which were pre-assigned to each study site before the start of the treatment. When the onset of ulcer was diagnosed endoscopically or the NSAID was changed to a different drug, the subjects were excluded from the study at that time point. To monitor the status of subject compliance to AG-1749 or gefarnate, the dosage (number of capsules) used for each was calculated and compared to that dispensed for each subject. The use of any medication that could affect the onset of gastric or duodenal ulcer, including corticosteroids, anti-platelet agents and anticoagulants, was prohibited during the course of the study.

### Outcomes and measurements

The primary endpoint was the recurrence of gastric or duodenal ulcers, defined as open ulcers (either active- or healing-stage) associated with a mucosal defect with whitish exudates measuring 3 mm or greater. All ulcers confirmed on endoscopy and reported from each study site were reconfirmed by the independent expert panel based on submitted films. The secondary endpoints were the development of gastric and/or duodenal hemorrhagic lesions as observed with endoscopy with or without hospital admission, treatment discontinuations due to lack of efficacy, gastric and/or duodenal mucosal damage as assessed with a modified Lanza score [13], and GI symptoms.

### Follow-up procedures

Endoscopy was scheduled every 12 weeks until 6 months of treatment and every 24 weeks after 6 months. Non-scheduled endoscopies were also performed if patients were suspected of having symptoms associated with ulcers or signs and symptoms indicative of GI bleeding.

Every 4 weeks, blood pressure was measured, clinical laboratory tests (chemistry, hematology, and urinalysis) were performed, compliance checks (returned tablet counts) conducted, and patients asked about any adverse effects they experienced. All patients were scheduled to receive the study treatments in a double-blind fashion until 6 months after the start of the study in the last enrollment. After the termination of the double-blind trial, patients in the 47 study sites were invited to participate in the follow-up study, in which all patients were treated once daily with lansoprazole 15 mg. If the onset of ulcer was confirmed on endoscopy in a patient, the patient discontinued his/her medication and antiulcer treatment such as full-dose PPI therapy was offered for ulcer healing.

### Statistical analysis

In an earlier study by Graham et al. [7], the recurrence rate was shown to be 20% in the lansoprazole group versus 49% in the placebo group, leading to a reduction of about 60% in the risk of ulcer recurrence in patients receiving lansoprazole. Also, a recent meta-analysis [14] suggested that PPIs may be associated with more potent anti-secretory effects in Asians than in Caucasians. Thus, using a conservative estimate, a 60% reduction in the risk of ulcer recurrence was assumed in Japanese patients receiving lansoprazole, equivalent to that in Caucasians, with an estimated annual ulcer recurrence of 8% (20% × 0.4 = 8%). On the other hand, to date, no data are available for prevention of ulcer recurrence during NSAID therapy with the reference drug gefarnate. In this respect, an earlier study by Agrawal et al. [15] evaluating misoprostol versus sucralfate (a close counterpart to gefarnate) given 3 months in patients with a history of NSAID-associated ulcer reported a recurrence rate of 16% in the sucralfate-treated group, from which, however, no definitive conclusions can be drawn regarding the prophylactic effect of sucralfate due to lack of a placebo-controlled arm in the study. Assuming that sucralfate (or gefarnate) should be significantly less potent than misoprostol or lansoprazole in prophylactic efficacy against ulcer recurrence, we estimated gefarnate in this study would reduce the rate of ulcer recurrence by 15% compared to placebo, leading to an annual ulcer recurrence of 17% (20% × 0.85).

This estimation meant that the hazard ratio (HR) of the lansoprazole-treated group relative to the gefarnate-treated group was 0.4475 under an exponential assumption on event distributions. The study required a total of 66 ulcer events (endpoints) for the two treatment groups to ensure a statistical power of 90% using a log-rank test with a two-sided alpha of 5%. To observe 66 events, the study required enrollment of 301 patients for each treatment group at randomization for a total of 602 patients, assuming a mean follow-up duration of 6 months and a 6-month dropout rate of 15%.

One interim analysis was planned in advance for the Independent Data Monitoring Committee to perform when half of the required number of ulcer events was observed. The O’Brien-Fleming boundary based on the information fraction of 0.5 was employed for an overall significance level of *α* = 0.05. To avoid unnecessary trial hazard to the patients assigned to either treatment, the investigators planned to discontinue the double-blind trial if the difference in the primary endpoint was shown to be significant in the interim analysis. However, this trial was prematurely terminated without doing the interim analysis based on the decision of the Independent Data Monitoring Committee.

The cumulative incidences of the primary and secondary endpoints were estimated by using the Kaplan–Meier method and compared between the treatment groups by using the log-rank test. For event-free cases, the event times were censored either at the point of the last endoscopy performed or at the point of early withdrawal. Multivariate Cox regression analyses were also performed to adjust for the possible effect of baseline variables on event times. The final analyses were conducted for the full analysis set (FAS), defined as all patients who were randomized and received one or more doses of the study medication. In the survival analysis, the patients at risk were defined as all event-free FAS patients who had at least one post-randomization assessment with endoscopy.

Differences in subjects demographics (Table 1) and adverse events (Table 5) between the lansoprazole and gefarnate groups were tested for significance by using the χ^2^ test, except mean age, mean duration of prior NSAID and compliance rate (all in Table 1) were tested by *t* test.

**Table 1:** Demographic and baseline characteristics of Japanese patients randomized to treatment

	Lansoprazole (*n* = 185)	Gefarnate (*n* = 181)	*P* value
Mean age (SD, years)	62.8 (11.72)	63.7 (11.05)	0.4501
Sex			0.7811
Male	73 (39.5)	74 (40.9)	
Female	112 (60.5)	107 (59.1)	
Current smoker	55 (29.7)	64 (35.4)	0.2504
Alcohol consumption	63 (34.1)	67 (37.0)	0.5538
Mean duration (SD) of prior NSAID (months)^a^	21.8 (14.87)	22.1 (14.37)	0.8445
Status of concomitant NSAID use			0.7018
Loxoprofen sodium hydrate	72 (38.9)	76 (42.0)	
Meloxicam	30 (16.2)	30 (16.6)	
Diclofenac sodium	22 (11.9)	27 (14.9)	
Etodolac	24 (13.0)	20 (11.0)	
Others	37 (20.0)	28 (15.5)	
Underlying disease^b^			
Rheumatoid arthritis	75 (40.5)	76 (42.0)	0.4174
Osteoarthritis	64 (34.6)	66 (36.5)	0.7087
Low back pain	6 (3.2)	8 (4.4)	0.5574
Others	85 (45.9)	72 (39.8)	0.2333
*H. pylori* status			0.3966
Positive	93 (50.3)	99 (54.7)	
Negative	92 (49.7)	82 (45.3)	
CYP2C19 polymorphism			0.5081
PM	32 (17.3)	35 (19.3)	
EM	137 (74.1)	125 (69.1)	
Mean compliance rate (SD)			
Study drug	97.5 (11.1)	97.9 (5.1)	0.6570
NSAID therapy	93.1 (10.4)	93.5 (6.3)	0.6558

Data are presented as numbers (and % of total) except where otherwise indicated

Unknown in 37 patients for whom consent was not obtained for the CYP2C19 polymorphism test

*PM* poor metabolizers, *EM* extensive metabolizers

^a^Those who reported taking NSAIDs for >3 years prior to the start of the study medication were construed as having taken them for 3 years

^b^Some patients were included in more than one disease category. “Others” include treatments such as lumbar spinal stenosis or intervertebral disc hernia

Analyses were conducted by using SAS software (version 9.1.3; SAS Institute Inc., Cary, NC, USA). One and the same statistician (SM) had full access to all the trial data and conducted statistical analyses independently of the sponsoring company.

### Detection of infection and CYP2C19 polymorphisms

*H. pylor*i infection status was determined for each patient by using an E-plate Eiken *H. pylori* antibody assay kit (Eiken chemical Co., Ltd.) at a central laboratory. Patients were judged to be “negative” if the antibody level was <10 U/ml. For CYP 2C19 pharmacogenomics analysis, the whole blood samples were collected from patients who gave separate informed consent for CYP2C19 polymorphism analysis. Polymorphisms and types of CYP2C19 metabolization (extensive or poor) were determined for all consenting subjects by the PCR–RFLP method or fluorescent correlation spectroscopy [16] at a central laboratory.

### Role of the funding source

Takeda Pharmaceutical Company and its contractor provided all financial and material support for the study design, data collection, data analysis, data interpretation, and preparation and review of manuscripts. The Sponsor was also responsible for consultations with the authors and the members of this study group about the study design and monitoring of the study. The principal investigator (KS) was responsible for the study design as well as for preparation of the manuscript. All co-authors reviewed the manuscript and necessary revisions were made to accommodate their suggestions and opinions.

## Results

### Study patients

This prospective, double-blind, randomized, active-controlled trial with an open-label 6-month follow-up study was conducted at a total of 99 healthcare institutions in Japan, in accordance with the principles of good clinical practice and the Declaration of Helsinki, from April 2007 to October 2009 (inclusive of the follow-up study). Based on the results of the preceding LDA study [12], the Independent Data Monitoring Committee made the decision to terminate the double-blind part of the study early. The results presented here are based on the completed and analyzed final data.

**Fig. 1:**
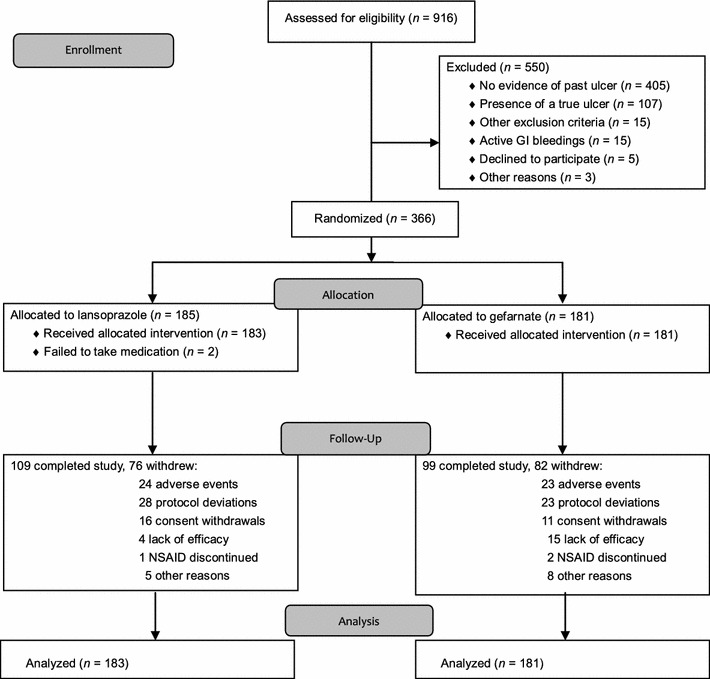
Patient disposition in this trial (2010 CONSORT flow diagram). As noted in the manuscript, the study was prematurely terminated for ethical reasons before accrual of the expected number of patients. *GI* gastrointestinal

Figure 1 shows the flow diagram in this trial. Of the 916 patients enrolled, 366 patients were randomized, while the remaining 550 patients were excluded primarily because they were not confirmed to meet the inclusion criteria. Of the 366 patients randomized, 185 were assigned to receive lansoprazole and 181 to receive gefarnate. Of the 185 patients assigned to lansoprazole, two patients were not given the study medication because one patient voluntarily discontinued the study medication and the other was lost to follow-up after the initial prescription.

The FAS population comprised a total of 364 patients, with 183 and 181 patients in the lansoprazole group and the gefarnate group, respectively. The numbers of withdrawals were similar between the treatment groups, with 76 (41.1% of 185 randomized patients) in the lansoprazole group and 82 (45.3%) in the gefarnate group. The most frequent reasons for withdrawal were protocol deviations (including failure to take the medication), which occurred in 28 (36.8%) patients in the lansoprazole group and in 23 (28.0%) patients in the gefarnate group, followed by adverse reactions and consent withdrawals in 24 (31.6%) and 16 (21.1%) patients, respectively, in the lansoprazole group, and in 23 (28.0%) and 11 (13.4%) patients, respectively, in the gefarnate group. Additionally, four patients in the lansoprazole group and 15 patients in the gefarnate group withdrew due to lack of efficacy or suspected ulcer-related symptoms/diagnoses. The median duration of follow-up was 6.6 months (range 0.0–22.2) for the lansoprazole group and 3.8 months (range 0.1–19.8) for the gefarnate group, with the follow-up being 2.8 months longer in the lansoprazole group, with many patients discontinuing gefarnate due to recurrence of gastric or duodenal ulcer. Compliance with the study medication and NSAID therapy was similarly high in the two treatment groups. There was no difference between the treatment groups in the frequency distribution of baseline variables (Table 1).

### Efficacy

**Fig. 2:**
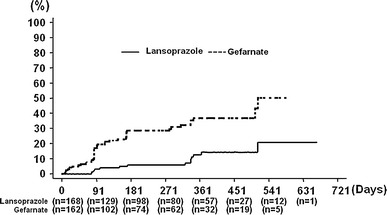
Kaplan–Meier estimates of the cumulative incidence of gastric or duodenal ulcers in the treatment groups

In the FAS population, the cumulative number of gastric or duodenal ulcer recurrences, i.e., primary endpoint, at the end of the study was 15/183 (8.2%) in the lansoprazole group and 46/181 (25.4%) in the gefarnate group (Table 2). The cumulative recurrences at days 91, 181, and 361 from the start of the study were estimated as 3.3% (95% CI 0.45–6.18), 5.9% (95% CI 1.87–9.83), and 12.7% (95% CI 5.85–19.59), respectively, for the lansoprazole group, compared to 18.7% (95% CI 12.27–25.07), 28.5% (95% CI 20.69–36.39), and 36.9% (95% CI 27.51–46.35), respectively, for the gefarnate group (Fig. 2). The HR of the lansoprazole group relative to the gefarnate group was 0.2510 (95% CI 0.1400–0.4499), which implied a 74.9% risk reduction, and the difference was highly significant (log-rank test, *P* < 0.0001) (Table 2).

**Table 2:** Effect of lansoprazole on each component of the primary and secondary endpoints

	Lansoprazole^a^ (*n* = 183)	Gefarnate^b^ (*n* = 181)	Hazard ratio (95% CI)	*P* value^c^
Number at risk at baseline^d^	168	162		
Primary endpoint				
Gastric or duodenal ulcer	15	46	0.2510 (0.1400–0.4499)	<0.0001
Secondary endpoints				
Gastric/duodenal ulcer and/or hemorrhagic lesion	15	52	0.2196 (0.1235–0.3904)	<0.0001
Gastric/duodenal ulcer, hemorrhagic lesion and/or treatment discontinuation due to lack of efficacy	18	65	0.2158 (0.1279–0.3640)	<0.0001
Component^e^				
Gastric or duodenal ulcer	13	42		
Hemorrhagic lesion	0	4		
Treatment discontinuation due to lack of efficacy	3	13		
Gastric or duodenal ulcer and hemorrhagic lesion	2	4		
Hemorrhagic lesion and treatment discontinuation due to lack of efficacy	0	2		

^a^Patients received lansoprazole 15 mg daily

^b^Patients received gefarnate 50 mg twice daily

^c^Log-rank test

^d^The number of patients at risk included all full analysis set patients who received at least one endoscopy assessment post-randomization, and had no acute-stage or healing-stage gastric or duodenal ulcer as confirmed by the Independent Adjudication Committee

^e^The ‘component’ section is intended to indicate the components of the endpoints given above and not the endpoints themselves. Hence, the hazard ratios and *P* values have not been calculated

As to the secondary endpoints (Table 2), the risk of developing gastric/duodenal ulcers or hemorrhagic lesions in the lansoprazole group was significantly lower than that in the gefarnate group (log-rank test, *P* < 0.0001). Similarly, the risk of having gastric/duodenal ulcers, hemorrhagic lesions, or treatment discontinuations due to lack of efficacy was significantly lower in the lansoprazole group than in the gefarnate group (log-rank test, *P* < 0.0001).

The magnitude of risk reduction in gastric or duodenal ulcers (primary endpoint) was generally stable for all subgroups as defined by each baseline variable (Table 3). The analyses in both *H. pylori*-positive and -negative subgroups showed ulcer risk reductions, with a HR of 0.1798 (95% CI 0.0740–0.4369; *P* < 0.0001) and 0.3327 (95% CI 0.1504–0.7361), respectively, in each of the subgroups in the lansoprazole group as compared to the gefarnate group. Furthermore, the risk reduction in terms of HR was estimated as 0.272 (95% CI 0.146–0.504; *P* < 0.0001 by a Wald test) after adjustment for the baseline variables, *H. pylori* status, CYP2C19 polymorphism, age, sex, smoking, alcohol consumption, and concomitant use of anticoagulants in a multivariate Cox regression analysis (Table 4).

**Table 3:** Analysis of subgroups as defined by each baseline variable

Baseline characteristic	Recorded number of patients with gastric or duodenal ulcer		Cox regression analysis	
	Lansoprazole	Gefarnate	Hazard ratio (95% CI)	*P* value
*H. pylori* status				
Positive	6/81^a^	27/87^b^	0.1798 (0.0740–0.4369)	<0.0001
Negative	9/87^a^	19/75^b^	0.3327 (0.1504–0.7361)	0.0044
CYP2C19				
PM	4/30^c^	7/32^d^	0.4675 (0.1360–1.6070)	0.2167
EM	10/124^c^	30/112^d^	0.2408 (0.1176–0.4929)	<0.0001
Age (years)				
25–64	7/86	20/83	0.2254 (0.0946–0.5370)	0.0002
65–85	8/82	26/79	0.2766 (0.1251–0.6112)	0.0007
Smoking status				
Yes	5/48	19/60	0.2262 (0.0840–0.6088)	0.0014
No	10/120	27/102	0.2649 (0.1281–0.5481)	0.0001
Alcohol consumption				
Yes	5/57	16/63	0.2873 (0.1048–0.7877)	0.0096
No	10/111	30/99	0.2377 (0.1161–0.4866)	<0.0001
Underlying disease				
Rheumatoid arthritis				
Yes	7/68	21/67	0.2342 (0.0993–0.5522)	0.0003
No	8/100	25/95	0.2664 (0.1201–0.5913)	0.0005
Osteoarthritis				
Yes	4/59	17/58	0.1904 (0.0640–0.5665)	0.0009
No	11/109	29/104	0.2860 (0.1426–0.5734)	0.0002

Data are presented as numbers at risk

At risk: the number of patients at risk included all full analysis set patients who had at least one post-randomization endoscopy assessment, and had no acute-stage or healing-stage gastric or duodenal ulcer as confirmed by the Independent Adjudication Committee

Results of Cox regression analyses in lansoprazole or gefarnate group, respectively, between each group indicated (*H. pylori*-positive vs. -negative and CYP2C19 PM vs. EM); hazard ratio (95% CI) and *P* value

*PM* poor metabolizers, *EM* extensive metabolizers

^a^ *H. pylori*-positive vs. -negative; 0.6306 (0.2240–1.7750), *P* = 0.3825

^b^ *H. pylori*-positive vs. -negative; 1.1418 (0.6338–2.0571), *P* = 0.6588

^c^PM vs. EM; 0.6890 (0.2160–2.1977), *P* = 0.5291

^d^PM vs. EM; 1.2611 (0.5520–2.8812), *P* = 0.5821

**Table 4:** Results of multivariate Cox regression analysis using baseline variables

Baseline characteristics	Direct estimation	Multivariate analysis	
		Hazard ratio (95% CI)	*P* value
Treatment group	Lansoprazole/gefarnate	0.271 (0.146–0.504)	<0.0001
*H. pylori* status	Positive/negative	0.986 (0.565–1.721)	0.9616
CYP2C19	PM/EM	1.031 (0.518–2.054)	0.9308
Age	10 years’ increase	1.189 (0.913–1.548)	0.1987
Sex	Male/female	0.978 (0.524–1.827)	0.9455
Smoking status	Yes/no	1.5 (0.815–2.758)	0.1925
Alcohol consumption	Yes/no	0.774 (0.412–1.457)	0.4277

*PM* poor metabolizers, *EM* extensive metabolizers

We also analyzed sites of recurrent ulcers to examine whether they recurred in similar sites to the scars observed at the start of the study. In 26 (42.6%) of these patients, ulcer recurrence was observed in similar sites to the scars seen at the start of the study.

GI damage as assessed by a modified Lanza score [13] from the start of treatment tended to improve in the lansoprazole group but to worsen in the gefarnate group, throughout the course of treatment, with a significant difference seen between the treatment groups from baseline to 6 months after the start of treatment (Supplemental Fig. 1a, 1b).

In the FAS population, the cumulative number of patients who developed gastric or duodenal hemorrhagic lesions at the end of the study was two of the 183 patients in the lansoprazole group compared to 10 of the 181 patients in the gefarnate group. The cumulative incidence rate was calculated by the Kaplan–Meier method (Supplemental Fig. 2), and the risk of hemorrhage was shown to be significantly lower in the lansoprazole group than in the gefarnate group. Bleeding ulcers occurred in two patients in the lansoprazole group and in three in the gefarnate group.

Of the 366 patients randomized to lansoprazole or gefarnate in this trial, 113 who had received lansoprazole or gefarnate were included in an open-label follow-up study to examine outcomes after another 24 weeks of treatment with lansoprazole, in addition to NSAID therapy. During this open-label follow-up trial period, five gastric or duodenal ulcer recurrences were observed in the continuous lansoprazole group (*n* = 73) and two recurrences were observed in the 40 patients who had taken gefarnate in the double-blind study and were subsequently included in the open-label study (Supplemental Table 1).

## Adverse events

With respect to adverse events observed in the double-blind study period (Table 5), nasopharyngitis and diarrhea were ≥5% more frequent in the lansoprazole group than in the gefarnate group, while reflux esophagitis was noted ≥5% more frequently in the gefarnate group. Serious adverse drug reactions were seen in two patients in the lansoprazole group (duodenitis and ovarian neoplasm) versus one in the gefarnate group (cardiac failure). Four deaths occurred total: two deaths in the lansoprazole group due to necrotizing pancreatitis and myocardial infarction, whose causal relationship to lansoprazole was denied, and two deaths in the gefarnate group due to pancreatic cancer and cardiac failure. The relationship between gefarnate and the death from pancreatic cancer was denied, and there was no definite causal relationship established between gefarnate and the death due to cardiac failure. No deaths occurred in the follow-up study period.

**Table 5:** Frequency of adverse events

Adverse events observed in the double-blind period	Lansoprazole (*n* = 183)	Gefarnate (*n* = 181)	*P* value
All adverse events	154 (84.2) [121.2]	125 (69.1) [127.7]	0.0006
Causal relationship to drug not deniable	28 (15.3) [22.0]	28 (15.5) [28.6]	0.9643
Leading to treatment discontinuations	29 (15.8)	23 (12.7)	0.3920
Serious adverse events	29 (15.8) [22.8]	17 (9.4) [17.4]	0.0638
Causal relationship to drug not deniable	2 (1.1) [–]	1 (0.6) [–]	0.5685
Deaths	2 (1.1)	2 (1.1)	0.9918
Adverse events reported in at least 3% of total in each group			
Nasopharyngitis	57 (31.1) [44.9]	42 (23.2) [42.9]	0.0769
Diarrhea	19 (10.4) [15.0]	–	<0.0001
Fall	12 (6.6) [9.4]	10 (5.5) [10.2]	0.6793
Constipation	10 (5.5) [7.9]	10 (5.5) [10.2]	0.9798
Eczema	10 (5.5) [7.9]	–	0.0014
Osteoarthritis	8 (4.4) [6.3]	6 (3.3) [6.1]	0.6001
Reflux esophagitis	–	12 (6.6) [12.3]	0.0003
Hypertension	7 (3.8) [5.5]	8 (4.4) [8.2]	0.7753
Contact dermatitis	–	7 (3.9) [7.2]	0.0072
Nausea	6 (3.3) [4.7]	–	0.0140
Foot tinea	6 (3.3) [4.7]	–	0.0140
Pneumonia	6 (3.3) [4.7]	–	0.0140
Back pain	6 (3.3) [4.7]	–	0.0140
Elevated blood creatine phosphokinase levels	6 (3.3) [4.7]	8 (4.4) [8.2]	0.5713
Rheumatoid arthritis	–	6 (3.3) [6.1]	0.0130

Table data are numbers (%) of patients in whom an event occurred at least one time during the trial. Numbers in brackets for adverse events are incidence rates in person-years

During the entire study, including the follow-up period, adverse events leading to treatment discontinuations were seen in 35 of the 223 patients treated with lansoprazole (Supplemental Table 2), where diarrhea was the most frequent of all events reported.

## Discussion

Risk factors for NSAID ulcer include advanced age [1], a history of ulcers or GI bleeding [3], and concomitant use of anticoagulants [4]. It is of note that the present study represents the first to provide evidence for the prophylactic effect of lansoprazole 15 mg against NSAID-associated ulcer recurrence in Japanese patients with a definite history of ulcer. Pharmacological inhibition of cyclooxygenase (COX) by NSAIDs is shown to decrease COX-derived prostaglandin production, suggesting that decreased endogenous prostaglandin production in the gastric mucosa may result in the disruption of mucosal resistance to gastric acid, thereby inducing the onset of gastric or duodenal ulcers as adverse events. In this regard, both lansoprazole and sucralfate, a drug of the same class with gefarnate, are shown to produce overall increases in duodenal mucosal turnover and transforming growth factor-alpha (TGF-α) levels, as well as in epidermal growth factor receptor (EGF-r) levels [17]. In light of these findings, therefore, the greater protection against NSAID-associated ulcer recurrence seen with low-dose lansoprazole than with gefarnate may be accounted for by the potent acid-inhibitory effect of low-dose lansoprazole.

Given that no drug has been proven to be effective for prevention of gastric or duodenal ulcer associated with long-term NSAID therapy in Japan and that it is unethical to conduct a placebo-controlled trial in patients at high risk of developing gastric or duodenal ulcers, the current study was designed to compare the efficacy of lansoprazole 15 mg once daily and gefarnate 50 mg twice daily. Gefarnate was included as an active-control, since it is a cytoprotective anti-ulcer agent commonly used in Japan in reducing the risk for gastric and duodenal ulcers in daily medical practice. To minimize the risk to the patients enrolled in this trial, they were strictly assessed by endoscopic examination for eligibility. In addition, unlike most long-term clinical trials conducted to date in a similar patient population, frequent endoscopic examinations (every 3 or 6 months) were scheduled by the protocol to closely monitor the study subjects to ensure early detection of ulcer recurrence, similar to the LDA study [12].

As in the LDA study [12], this study also included both *H. pylori*-positive and -negative patients without any eradication therapy, given the relatively high background prevalence of *H. pylori* infection in Japan. There are a number of reasons that support the rationale for this approach.

First, although *H. pylori* eradication is generally recommended in most situations [18–20], trial results reported to date are not consistent. One study [21] showed *H. pylori* eradication was less effective than omeprazole therapy in preventing recurrent GI bleeding, which was observed in 18.8% of patients in whom *H. pylori* had been eradicated versus 4.4% of patients receiving omeprazole therapy after 6 months of treatment. An 8-week, parallel group, prospective study [22] also showed that *H. pylori* infection promoted NSAID ulcer healing with either ranitidine (66%) or lansoprazole (74 and 50% for 15 and 30 mg). *H. pylori* eradication reduced the risk of NSAID ulcers especially in NSAID-naïve patients with the OR being 0.26, while it did not lead to a significant reduction of risk in those with a history of concurrent NSAID therapy (OR 0.95) [23].

Analyses of both *H. pylori*-positive and -negative subgroups showed ulcer risk reductions associated with study drug treatment, with these reductions being greater in *H. pylori*-positive patients assigned to lansoprazole but not in *H. pylori*-positive patients assigned to gefarnate. This finding appears to support the usefulness of low-dose lansoprazole for preventing gastric or duodenal ulcers associated with NSAID therapy in Japan, where the prevalence of *H. pylori* infection is high [24]. Additionally, although more *H. pylori*-negative patients will need prophylaxis against NSAID ulcers in Japan, where *H. pylori* infection rate is predicted to gradually decrease [25], the study findings suggest the usefulness of low-dose lansoprazole in high-risk patients requiring long-term NSAID therapy, regardless of *H. pylori* status.

An analysis of the study data showed that lansoprazole significantly reduced the risk of ulcer recurrences by almost 75%. Although the recurrence of ulcers observed with endoscopy was assessed as the primary endpoint in this study, other clinical endpoints, such as GI bleeding or patient hospitalization, have also been compared between the treatment groups, because these true clinical outcomes are highly relevant in evaluating the drugs for efficacy. More patients in the gefarnate group developed gastric or duodenal hemorrhagic lesions and were hospitalized with serious adverse events leading to gastric or duodenal bleeding in this study (Supplemental Table 3). Thus, overall, lansoprazole was superior to gefarnate in all endpoints assessed in the study.

There are studies reporting an increase of some adverse events such as community-acquired pneumonia (CAP), diarrhea, and bone fracture with the use of PPIs [26, 27]. Of the adverse events reported in the double-blind study, those seen more frequently in the lansoprazole group than in the gefarnate group were nasopharyngitis and diarrhea, with none of these being severe. With regard to diarrhea, while a potential causal relationship with the study medication in the lansoprazole group was not denied in 4.4% of its occurrences (8/183 patients), all these events were not severe or widely different from the incidence and severity of diarrhea reported earlier with lansoprazole [28]. In the case of pneumonia, a causal relationship with the study medication was denied in all patients in this study, and analyses of long-term use or meta-analyses did not support the association between PPIs and CAP [26, 28].

This study has several limitations: high dropout rate leading to differences in duration of treatment with either study drug among the participants; the study incorporated the gefarnate group only as a control group but not as a placebo group, unlike earlier studies [5, 6]; and all statistical analyses were performed on a fewer number of patients than that pre-specified before the start of the study, due to premature termination of the study for ethical reasons before accrual of the expected number of patients. The estimates of the treatment effect may have been biased by early termination of the study and the small number of events observed in the study. A further limitation is that endoscopic ulcer occurrence, a surrogate endpoint, was evaluated as the primary endpoint in this study, with the hard (true) endpoint of hemorrhagic events being addressed only in terms of the number of patients who developed these events.

Nevertheless, the authors believe that this study has its own merits. First, it clearly demonstrated that low-dose lansoprazole protected against ulcer development associated with NSAID usage. A double-blind study by Graham et al. [7] showed an ulcer risk reduction of about 60% in *H. pylori*-negative users of NSAIDs including LDA. Similar to the previous study demonstrating an ulcer risk reduction of about 90% with low-dose lansoprazole [12], irrespective of *H. pylori* status, in LDA users, this study demonstrated a 75% ulcer risk reduction in NSAID users, with both studies suggesting a higher risk reduction than that reported in the study by Graham et al. Furthermore, this study suggested the possibility that low-dose lansoprazole provided more potent prophylaxis against ulcers in *H. pylori*-positive patients than did gefarnate, suggesting that *H. pylori* positivity in the study subjects may be among the factors that accounted for a greater ulcer risk reduction in this study than in the study by Graham et al. Second, in this study, the subjects were endoscopically followed up as often as every 3 months to provide rigorous data on endoscopic ulcer development in these patients. Third, a 6-month, long-term follow-up period was incorporated into the study to evaluate the safety and efficacy of low-dose lansoprazole, and of note, no such study has been conducted by others to date. Indeed, in the entire study, including the 6-month follow-up period, the cumulative incidence rate of gastric or duodenal ulcers was shown to be 14.4 and 19.6% in patients treated with lansoprazole at days 361 and 631 (36.9 and 50.3% in the gefarnate group in the double-blind study data), suggesting the efficacy of lansoprazole sustained over a period of 12 months or longer, which is the longest period reported to date showing the effectiveness and safety of PPIs to prevent NSAID ulcers in high-risk patients.

In Japan, where the society is growing increasingly aged, there will be an increased need for NSAID therapy in the management of diseases associated with debilitating pain in the elderly. Therefore, prevention and treatment of NSAID-induced ulcers continues to be an urgent and important issue. In this context, lansoprazole appears to have a major role to play, as it is shown to reduce the risk of gastroduodenal ulcers in high-risk patients who require long-term NSAID therapy for pain relief in such diseases as RA, osteoarthritis, and low back pain, while at the same time allowing such NSAID therapy to relieve pain and inflammation associated with these debilitating diseases.

### Electronic supplementary material

Below is the link to the electronic supplementary material.

**Supplemental Fig. 1**. Changes in GI damage from baseline as assessed by a modified Lanza score [13]. Gastric and/or duodenal mucosal damage was evaluated endoscopically and graded on a 0 (no damage) to 4+ (worst damage) scale for gastric mucosal damage (a), and 0–3+ for duodenal damage (b). Wilcoxon rank-sum test was not applicable for 0 and 24 month time point (†). Thin lines indicate SD. *: P<0.01, *: p<0.01. (JPEG 217 kb)

**Supplemental Fig. 2.** Kaplan-Meier estimates of the cumulative incidence of gastric or duodenal hemorrhagic lesions in the treatment groups. (JPEG 34 kb)

Supplementary tables (DOCX 19 kb)

## Appendix: Investigators in the Lansoprazole Ulcer Prevention Study Group (NSAID Therapy)

Akira Sagawa, Sagawa Akira Rheumatology Clinic, Sapporo, Hokkaido, Japan; Masanori Murakami, Hokkaido PWFAC Asahikawa Kosei General Hospital, Asahikawa, Hokkaido, Japan; Yoshiharu Amasaki, Tonan Hospital, KKR Sapporo Medical Center, Sapporo, Hokkaido, Japan; Kazuhide Tanimura, Tokeidai Hospital, Sapporo, Hokkaido, Japan; Naofumi Yamauchi, Kiyota Hospital, Sapporo, Hokkaido, Japan; Junichi Suzuki, KKR Sapporo Medical Center, Sapporo, Hokkaido, Japan; Yutaka Suzuki, Hokkaido Health Coop Ryokuai Hospital, Sapporo, Hokkaido, Japan; Susumu Asano, Asano Seikeigeka Clinic, Chitose, Hokkaido, Japan; Takao Kodera, Tohoku Kosei-Nenkin Hospital, Sendai, Miyagi, Japan; Shigeru Wakatsuki, Taihaku-Sakura Hospital, Sendai, Miyagi, Japan; Takeshi Ohki, Yuki Hospital, Ibaraki, Japan; Jun Fujisaki, Hitachi, Ltd. Hitachinaka General Hospital, Ibaraki, Japan; Takehiko Ayabe, Ayabe Medical Clinic, Ibaraki, Japan; Kentaro Sugano, Jichi Medical University Hospital, Tochigi, Japan; Shunroku Sugimoto, Sugimoto Hospital, Gunma, Japan; Fusanori Maeda, Hanyu General Hospital, Saitama, Japan; Fuminobu Shinozaki, Shimoshizu National Hospital, Chiba, Japan; Masahiro Yoshioka, Minamitama Hospital, Tokyo, Japan; Megumi Hiida, Machida Municipal Hospital, Tokyo, Japan; Yoshinori Takahara, Hoya Kosei Hospital, Tokyo, Japan; Tatsuyuki Hori, Tokyo National Hospital, National Hospital Organization, Tokyo, Japan; Naomi Uemura, National Center for Global Health and Medicine, Tokyo, Japan; Masao Matsumoto, Tokyo Kosei Nenkin Hospital, Tokyo, Japan; Junya Mibe, Tokyo Metropolitan Otsuka Hospital, Tokyo, Japan; Hisakuni Sekino, Sekino Hospital, Tokyo, Japan; Akio Yokoi, Tokyo Medical Center, National Hospital Organization, Tokyo, Japan; Yasuhide Imamura, Eisei Clinic, Tokyo, Japan; Shigeto Kiyokawa, Fujinomori Clinic, Tokyo, Japan; Yoshio Ohashi, Tokyo Ekimae-Building Clinic, Tokyo, Japan; Shinichi Wada, Wada Orthopedic Clinic, Tokyo, Japan; Toshio Inoue, Shonan Memorial Hospital, Kanagawa, Japan; Shigeto Toma, Sagamihara National Hospital, National Hospital Organization, Kanagawa, Japan; Jun Takatsuka, Sagamihara Chuo Hospital, Kanagawa, Japan; Satoshi Kiyama, Chuo-Rinkan Hospital, Kanagawa, Japan; Hiroshi Yoshida, Saiseikai Yokohamashi Nanbu Hospital, Kanagawa, Japan; Sadaaki Miyagawa, Miyagawa Hospital, Kawasaki, Kanagawa, Japan; Hideki Iizuka, Seji Otsuka, Koukan Clinic, Kawasaki, Kanagawa, Japan; Muneatsu Toshima, Niitsu Medical Center Hospital, Niigata, Niigata, Japan; Teiji Kontani, Komatsu Municipal Hospital, Komatsu, Ishikawa, Japan; Tomofumi Morita, Morita Hospital, Komatsu, Ishikawa, Japan; Koji Shinmura, Shinmura Hospital, Hakusan, Ishikawa, Japan; Shinichi Katsuo, Nittazuka Iryou Fukushi Center, Fukui, Fukui, Japan; Shinji Wakabayashi, Matsumoto Medical Center, Matsumoto, Nagano, Japan; Takahiro Kato, Murakami Memorial Hospital, Asahi University, Gifu, Gifu, Japan; Yasushi Suzuki, Gifu Prefectural General Medical Center, Gifu, Gifu, Japan; Hirofumi Nishimoto, Gifu Central Hospital, Gifu, Gifu, Japan; Mitsuo Yatsuzuka, Shizuoka Medical Center, Sunto-gun, Shizuoka, Japan; Toru Maeda, Nagoya Tokushukai General Hospital, Kasugai, Aichi, Japan; Koichiro Morishita, Yokkaichi Social Insurance Hospital, Yokkaichi, Mie, Japan; Toru Yamakawa, Yamada Red Cross Hospital, Ise, Mie, Japan; Hitoshi Yoshimura, Mie Prefectural Shima Hospital, Shima, Mie, Japan; Tateo Sugiyama, Kanai Hospital, Kyoto, Kyoto, Japan; Yasumasa Kondo, Horikawa Hospital, Kyoto, Kyoto, Japan; Jun Okada, Nozaki Tokushukai Hospital, Daito, Osaka; Shinya Ohashi, Shujiro Yazumi, Kitano Hospital, Osaka, Osaka, Japan; Kiyoshi Ashida, Osaka Saiseikai Nakatsu Hospital, Osaka, Osaka, Japan; Kiyotaka Okawa, Osaka City General Hospital, Osaka, Osaka, Japan; Tetsuhiko Nakamura, Nakamura Clinic, Sakai, Osaka, Japan; Naohide Takigawa, Koyukai Nishinomiya Kyoritsu Neurosurgical Hospital, Nishinomiya, Hyogo, Japan; Hiroyuki Ogawa, Nishinomiya Municipal Central Hospital, Nishinomiya, Hyogo; Tsukasa Matsubara, Mayflower Clinic, Kato, Hyogo, Japan; Koji Kuranobu, Tottori Red Cross Hospital, Tottori, Tottori, Japan; Hirokazu Hashiguchi, Sanin Rosai Hospital, Yonago, Tottori, Japan; Hirofumi Tamura, Kosei Hospital, Okayama, Okayama, Japan; Shiro Okamoto, Kure Kyosai Hospital, Kure, Hishoshima, Japan; Hideaki Imada, Higashihiroshima Medical Center, Higashihiroshima, Hiroshima, Japan; Koichiro Ihara, Kanmon Medical Center, National Hospital Organization, Shimonoseki, Yamaguchi, Japan; Yoshitaka Matsubara, Yohei Nakamura, Shuto General Hospital, Yanai, Yamaguchi, Japan; Masahiko Hiasa, Yagi Hospital Nakasu, Minami-Awaji, Hyogo, Japan; Tatsuhiko Henmi, Health Insurance Naruto Hospital, Naruto, Tokushima, Japan; Tomoki Inaba, Kagawa Prefectural Central Hospital, Takamatsu, Kagawa, Japan; Kuniichi Kawano, Takamatsu Municipal Hospital, Takamatsu, Kagawa, Japan; Koichi Kurahara, Matsuyama Red Cross Hospital, Matsuyama, Ehime, Japan; Tadahiko Aibara, Aibara Orthopedic Clinic, Matsuyama, Ehime, Japan; Shuji Yokota, Yokota Orthopedic Clinic, Matsuyama, Ehime, Japan; Atsuko Imai, Ichiban-cho Clinic for Rheumatic Diseases, Matsuyama, Ehime, Japan; Nobuo Takubo, Takubo Rheumatology/Orthopedics Clinic, Matsuyama, Ehime, Japan; Mitsuru Kajitani, Kochi Rehabilitation Hospital, Kochi, Kochi, Japan; Masahiro Otani, Koryo Hospital, Susaki, Kochi, Japan; Kenichi Kitaoka, Seihei Tamura, Shinsuke Kono, Susaki Kuroshio Hospital, Susaki, Kochi, Japan; Yoshinori Takei, Izumino Hospital, Kochi, Kochi, Japan; Takaaki Fukuda, Kurume University Medical Center, Kurume, Fukuoka, Japan; Norito Matsukuma, Kurume Daiichi Social Insurance Hospital, Kurume, Fukuoka, Japan; Seiji Yoshizawa, Yoshiteru Ueda, Munakata Medical Association Hospital, Munakata, Fukuoka, Japan; Naohiko Harada, Kyushu Medical Center, Fukuoka, Fukuoka, Japan; Takahiko Sannomiya, Sannomiya Orthopedic Clinic, Kurume, Fukuoka, Japan; Daisuke Matsunaga, Matsunaga Orthopedic Clinic, Onga-gun, Fukuoka, Japan; Masaaki Morooka, Morooka Orthopedic Clinic, Chikushi-gun, Fukuoka, Japan; Kosuke Hyakutake, Hyakutake Orthopedic Hospital, Saga, Saga, Japan; Toshiyuki Tsuruta, Tsuruta Orthopaedic Clinic, Ogi, Saga, Japan; Kiyoshi Migita, Nagasaki Medical Center, National Hospital Organization, Omura, Nagasaki, Japan; Michiharu Mihara, Kumamoto City Hospital, Kumamoto, Kumamoto, Japan; Katsuhiko Sakuma, Japanese Red Cross Kumamoto Hospital, Kumamoto, Kumamoto, Japan; Masayuki Yasuda, Yasuo Suenaga, Beppu Medical Center, Beppu, Oita, Japan; Etsuo Chousa, University of Miyazaki Hospital, Miyazaki, Miyazaki, Japan; Yoshihide Ushitani, Clinic Ushitani, Miyazaki, Miyazaki, Japan; Masataka Inakura, Inakura Hospital, Miyazaki, Miyazaki, Japan; Sho Nagai, Ebino Centro Clinic, Ebino, Miyazaki, Japan; Takemasa Matsuda, Kagoshima Red Cross Hospital, Kagoshima, Kagoshima, Japan.
