# Supplementary figures and images for: Lansoprazole for secondary prevention of gastric or duodenal ulcers associated with long-term non-steroidal anti-inflammatory drug (NSAID) therapy: results of a prospective, multicenter, double-blind, randomized, double-dummy, active-controlled trial
Source: J Gastroenterol. 2012 Mar 3;47(5):540–52. doi: 10.1007/s00535-012-0541-z (PMC3360874; doi:10.1007/s00535-012-0541-z)

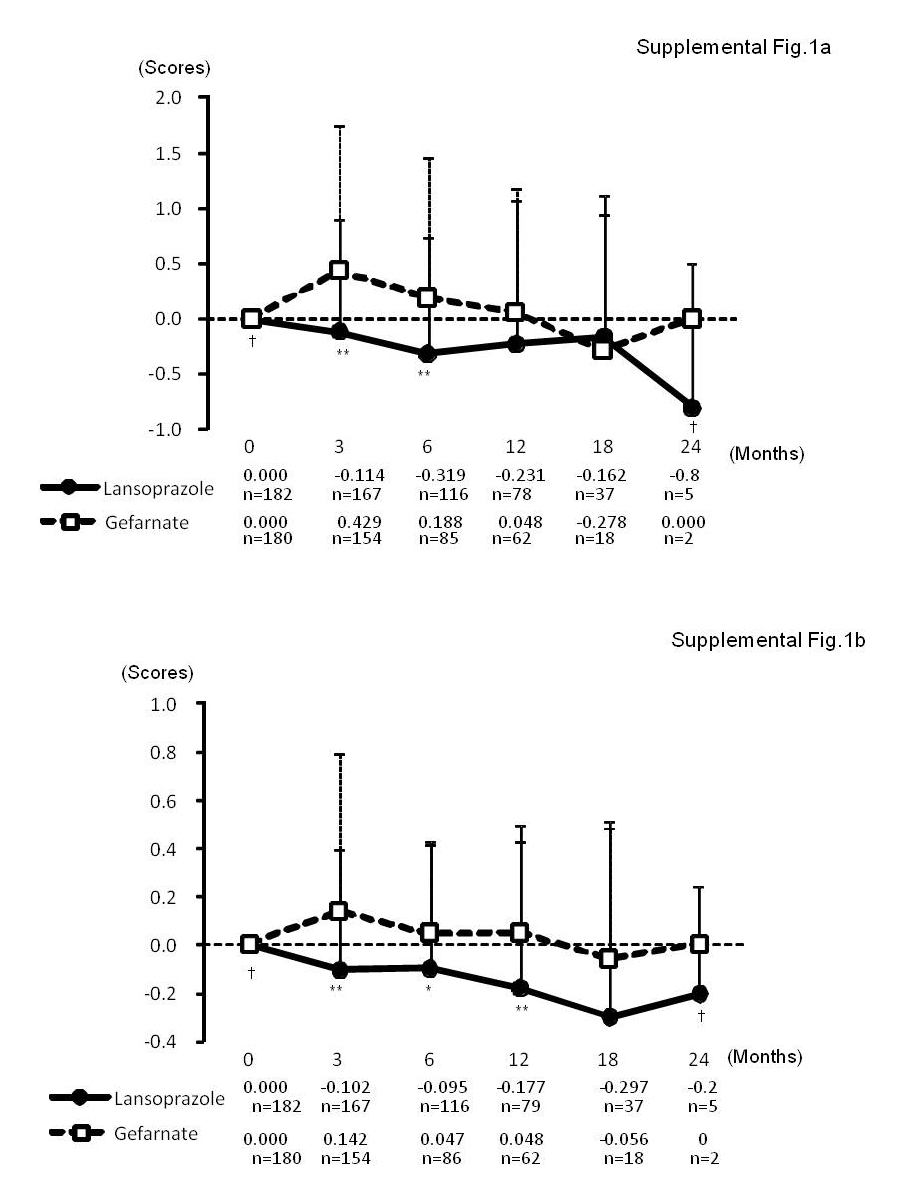

Supplement: Supplementary file 1 — Supplemental Fig. 1. Changes in GI damage from baseline as assessed by a modified Lanza score [13]. Gastric and/or duodenal mucosal damage was evaluated endoscopically and graded on a 0 (no damage) to 4+ (worst damage) scale for gastric mucosal damage (a), and 0–3+ for duodenal damage (b). Wilcoxon rank-sum test was not applicable for 0 and 24 month time point (†). Thin lines indicate SD. *: P<0.01, *: p<0.01. (JPEG 217 kb) [file 535_2012_541_MOESM1_ESM.jpg]

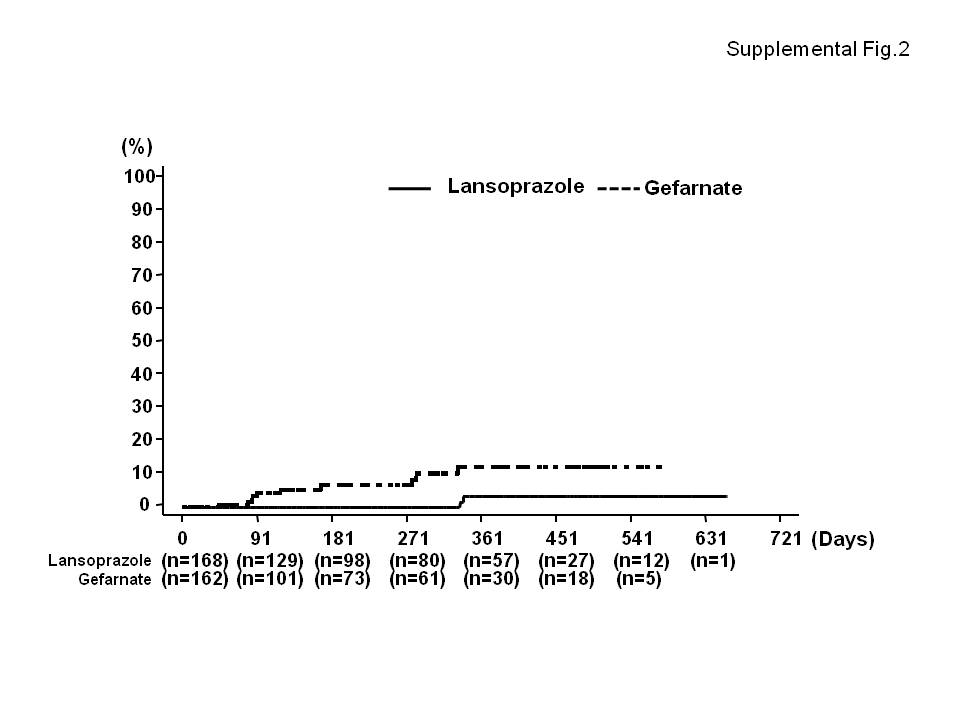

Supplement: Supplementary file 2 — Supplemental Fig. 2. Kaplan-Meier estimates of the cumulative incidence of gastric or duodenal hemorrhagic lesions in the treatment groups. (JPEG 34 kb) [file 535_2012_541_MOESM2_ESM.jpg]
