# Supplementary material for: Lansoprazole for secondary prevention of gastric or duodenal ulcers associated with long-term non-steroidal anti-inflammatory drug (NSAID) therapy: results of a prospective, multicenter, double-blind, randomized, double-dummy, active-controlled trial
Source: J Gastroenterol. 2012 Mar 3;47(5):540–52. doi: 10.1007/s00535-012-0541-z (PMC3360874; doi:10.1007/s00535-012-0541-z)
Supplement: Supplementary file 3 — Supplementary tables (DOCX 19 kb) [file 535_2012_541_MOESM3_ESM.docx]

# Supplemental Table 1. Baseline characteristics of Japanese patients in the follow-up study

|  | Lansoprazole to open-label lansoprazole (*n* = 73) | Gefarnate to open-label lansoprazole (*n* = 40) |
| --- | --- | --- |
| Mean age (SD) years | 63.6 (9.64) | 60.1 (12.90) |
| Sex |  |  |
| Male | 26 (35.6) | 17 (42.5) |
| Female | 47 (64.4) | 23 (57.5) |
| Current smoker | 19 (26.0) | 13 (32.5) |
| Alcohol consumption | 17 (23.3) | 19 (47.5) |
| Mean duration (SD) of prior NSAIDs (months) ^a^ | 22.294  (14.4862) | 20.810  (14.8235) |
| Status of concomitant NSAIDs use |  |  |
| Loxoprofen sodium hydrate | 26 (35.6) | 19 (47.5) |
| Meloxicam | 15 (20.5) | 7 (17.5) |
| Diclofenac sodium | 8 (11.0) | 7 (17.5) |
| Etodolac | 11 (15.1) | 5 (12.5) |
| Others | 13 (17.8) | 2 (5.0) |
| Underlying disease ^b^ |  |  |
| Rheumatoid arthritis | 29 (39.7) | 11 (27.5) |
| Osteoarthritis | 30 (41.1) | 13 (32.5) |
| Low back pain | 3 (4.1) | 0 (0.0) |
| Others | 26 (35.6) | 18 (45.0) |
| *H. pylori* status |  |  |
| Positive | 36 (49.3) | 25 (62.5) |
| Negative | 37 (50.7) | 15 (37.5) |
| CYP2C19 polymorphism |  |  |
| PM ^c^ | 16 (21.9) | 13 (32.5) |
| EM ^c^ | 53 (72.6) | 23 (57.5) |
| Mean compliance rate (SD) % |  |  |
| Study drug | 99.30 (1.150) | 98.88 (2.267) |
| NSAID therapy | 94.6 (1.34) | 92.8 (10.41) |
| Recurrence of gastric or duodenal ulcer | 5 (6.8) | 2 (5.0) |
| Data are presented as numbers (and % of total) except where otherwise indicated.  ^a^ Those who reported taking NSAIDs for >3 years prior to the start of the study medication were construed as having taken them for 3 years.  ^b^ Some patients were included in more than 1 disease category. “Others” include treatments such as Lumbar spinal stenosis or Intervertebral disc protrusion.  ^c^ PM = poor metabolizers,EM = extensive metabolizers; unknown in 40 patients for whom consent was not obtained for the CYP2C19 polymorphism test. | | |

**Supplemental Table 2.** Frequency of adverse events throughout the double-blind and the follow-up period

| Adverse events reported in patients who received lansoprazole throughout the double-blind and open-label study | Lansoprazole (*n* = 223) |
| --- | --- |
| All adverse events | 187 (83.9) |
| Causal relationship to drug not deniable | 36 (16.1) |
| Leading to treatment discontinuations | 35 (15.7) |
| Serious adverse events | 36 (16.1) |
| Causal relationship to drug not deniable | 2 (0.9) |
| Adverse events reported in at least 3% of total in each group |  |
| Nasopharyngitis | 64（28.7） |
| Diarrhea | 22 (9.9) |
| Fall | 19 (8.5) |
| Eczema | 11 (4.9) |
| Constipation | 11 (4.9) |
| Hypertension | 10 (4.5) |
| Insomnia | 10 (4.5) |
| Osteoarthritis | 8 (3.6) |
| Contact dermatitis | 8 (3.6) |
| Foot tinea | 8 (3.6) |
| Muscle spasm | 7 (3.1) |
| Elevated blood triglyceride levels | 7 (3.1) |
| Back pain | 7 (3.1) |

Table data are numbers (%) of patients in whom an event occurred at least 1 time during the trial.

**Supplemental Table 3.** Cases of GI tract bleeding observed during the double-blind study

Cases of GI tract bleeding observed during treatment with low-dose lansoprazole

| Age (y) | Sex | Bleeding Status | Site of Bleeding | Cause | Hospitalization |
| --- | --- | --- | --- | --- | --- |
| 66 | Female | Adherent blood clot | Stomach | Gastric ulcer | No |
| 52 | Male | Oozing hemorrhage | Stomach | Gastric ulcer | No |

Cases of GI tract bleeding observed during treatment with gefarnate

| Age (y) | Sex | Bleeding Status | Site of Bleeding | Cause | Hospitalization |
| --- | --- | --- | --- | --- | --- |
| 52 | Female | Adherent blood clot | Stomach | Petechial hemorrhagic erosion | No |
| 47 | Male | Adherent blood clot | Duodenum | Duodenal ulcer | No |
| 82 | Male | Adherent blood clot | Stomach | Polyps | No |
| 68 | Male | Adherent blood clot | Stomach | Erosion | No |
| 65 | Female | Adherent blood clot | Stomach | Acute hemorrhagic erosion | No |
| 78 | Male | Adherent blood clot | Stomach | Acute gastric mucosal lesion | No |
| 64 | Female | Adherent blood clot | Stomach | Gastroduodenal ulcer | No |
|  |  | Adherent blood clot | Duodenum | Gastroduodenal ulcer | No |
| 68 | Female | Adherent blood clot | Stomach | Gastric ulcer (2.5 mm) | No |
| 56 | Male | Adherent blood clot | Stomach | Acute gastric mucosal lesion | No |
| 73 | Female | Adherent blood clot | Stomach | Gastric ulcer | Yes |
